# Intermixing of Unoccupied States of Metal Phthalocyanine Chains Assembled on Au(110)

**DOI:** 10.3390/nano14020158

**Published:** 2024-01-11

**Authors:** Abhishek Kumar, Maria Grazia Betti, Carlo Mariani, Manvendra Kumar, Pierluigi Gargiani, Cristian Soncini, Maddalena Pedio

**Affiliations:** 1 Istituto Officina dei Materiali-Consiglio Nazionale delle Ricerche, Basovizza SS-14, Km 163.5, 34012 Trieste, Italy; abhishek.e9363@cumail.in (A.K.); kmanav@gmail.com (M.K.); cristian.soncini@ifn.cnr.it (C.S.); 2 Department of Physics, University of Trieste, 34127 Trieste, Italy; 3 Dipartimento di Fisica, Università di Roma “La Sapienza”, Piazzale Aldo Moro 5, 00185 Roma, Italy; maria.grazia.betti@roma1.infn.it (M.G.B.); carlo.mariani@uniroma1.it (C.M.); 4 Department of Physics, Institute of Science, Shri Vaishnav Vidyapeeth Vishwavidyalaya, Ujjain Road, Indore 453111, India; 5 ALBA Synchrotron Light Source, Carrer de la Llum 2-26, 08290 Barcelona, Spain; pgargiani@cells.es; 6 Istituto Officina dei Materiali-Consiglio Nazionale delle Ricerch, V. A. Pascoli s.n.c., 06123 Perugia, Italy

**Keywords:** phthalocyanine, inverse photoemission spectroscopy, X-ray absorption spectroscopy, self-assembled molecules

## Abstract

A detailed inverse photoemission study unveils the unoccupied electronic structure induced by the adsorption of CuPc and CoPc phthalocyanines on Au(110) reconstructed channels. The different behavior in the two systems is related to the different intermixing of orbitals with the underlying gold states. Broadening of the density of states at the Fermi level is detected after CoPc adsorption, absent in the case CuPc. A detailed comparison with the element-selective X-ray absorption spectroscopy enlightens and complements the IPES results and confirms a surface-driven intermixing of the CoPc orbitals involved in the interaction, with the out-of-plane Co 3d_z_^2^ orbital strongly hybridized with the gold electronic states. Moreover, the contribution of the 3d empty states to the IPES data is reported for FePc, CoPc, and CuPc thin films.

## 1. Introduction

Transition metal phthalocyanines (MN_8_C_32_H_16_, MPcs) are planar tetrapyrroles formed of a conjugated macrocycle coordinating a central metallic ion, suitable for a variety of applications, including spintronics, field-effect transistors, and other electronic devices [1,2,3]. 

MPcs can form highly ordered supramolecular architectures on metal surfaces, with bonds, weak enough to guarantee high stability at room temperature (RT) [4,5,6], which is crucial for their technological use. The properties of the molecular films used in the applications are influenced by surface/interface details, which modulate the supramolecular organization. Detailed knowledge of the interface electronic structure is a prerequisite for insightful materials design and to exploit the potential of these molecules for impactful applications. MPcs with a magnetic central ion can be used as a chemical cage for anchoring the magnetic ions to a metal surface. These assembled metal networks can be easily manipulated, and the spin state and magnetic response can be tuned by modifying the molecular conformation and/or the molecule–substrate interaction [6]. The ability to control and manipulate the individual spin of the metal atoms and the coupling of single spins to their environment are at the basis of perspective quantum technologies [7,8,9,10]. A regular spin network can be formed on suitable templates, and the strength of the molecule–substrate interactions regulates the interfacial and electronic properties of metal-supported metalorganic molecules. 

The electro-magnetic properties of self-assembled MPcs are significantly influenced by the nature of the central metal ion. The occupancy of its 3d levels that contribute to the frontier orbitals is related to a complex multiplet structure in the presence of the D_4h_ crystal field (see SI Table S1 and Figure S1), notably influencing the spin of central metal ions. In particular, CoPc and CuPc are both paramagnetic, with an S = ½ spin state, a singly occupied molecular orbital (SOMO), and a counterpart, the single unoccupied molecular orbital (SUMO) [3,11]. 

The Au(110) surface is highly anisotropic, presenting a 1 × 2 missing row reconstruction. MPc layers in contact with Au(110) have been deeply investigated in the literature through diffraction techniques, namely low-energy electron diffraction (LEED) and grazing incidence X-ray diffraction (GIXRD), that reveal (5 × 5) and (5 × 3) reconstructions for CuPc [5] or (5 × 7) for FePc at different film coverages [12] and strong structural perturbations in the gold. The molecules lie flat on the substrate up to the single-layer completion, as proved by XAS [4,5,6] and Scanning Tunnelling Microscopy (STM), with the MPc arranged compact chains along the [1$\bar{1}$0] Au(110) direction [5,12]. Moreover, these systems have been studied via photoemission [4,5,13,14], linear-polarized XAS [15], and X-ray Magnetic circular Dichroism (XMCD) [6]. 

Co L_3_ XAS of CoPc establishes the contribution of the a_1g_ state, due to the 3d_z_^2^ (orthogonal to the molecular plane) at the edge of the spectrum [15]. In CuPc XAS, the main contribution of the first peak at the Cu L_3_ edge has an in-plane direction and b_1g_ symmetry. Notably, XMCD reveals a total quenching of CoPc magnetic moment on Au(110) for coverages up to a single layer, at the variance of CuPc, suggesting a stronger interaction of CoPc with the underlying gold atoms.

The present work is focused on an inverse photoemission spectroscopy (IPES) investigation to obtain insights into the unoccupied electronic states in the interaction process of MPc/Au(110). An IPES spectrum reveals the density of states (DOS) of the unoccupied levels through the photons emitted by the radiative relaxation of electrons injected into the unoccupied states of the sample. The IPES data are here compared with element-sensitive XAS results, often used in the literature to probe the unoccupied states, though the presence of core holes after X-ray absorption leads to different final states in the two techniques [16]. 

Detailed knowledge of the changes induced by adsorption in molecular-occupied and -unoccupied electronic states is crucial due to their key role as pathways for charge transfer (CT) and injection into the electrodes of the related devices. The advantage of IPES consists of providing the DOS of the unoccupied electronic structure, in good approximation. Moreover, the organic unoccupied states present a high sensitivity to state hybridizations. This is due to their high delocalization around the macrocycle, as discussed in ref. [17], and a higher energy separation, in comparison with the filled states, making IPES a sensitive technique for molecular perturbations. Additional important information provided by IPES is the empty level alignment and the identification of the unoccupied frontier orbital, crucial for electron transport in application.

IPES data have been published for tetrapyrrole thin films [18,19,20,21,22,23,24,25,26,27,28], and the unoccupied density of states of the CuPc adlayer on metal and semiconductor surfaces [18,19,20,21,22,23,24] or nanoparticles [25] is the most studied MPc and well characterized in the literature. The CoPc IPES literature is not as rich (see [20,21,28] and references therein).

Differences in the bond intermixing of the CuPc and CoPc with the Au(111) surface have been found based on combined photoemission-IPES, revealing opposite CTs for CoPc and CuPc. In the first case, electrons are transferred from gold to the adsorbate, while in the latter, the transfer occurs oppositely [19]. This phenomenon is related to different energy level alignments and interface dipoles and has been ascribed to the 3d specific configuration in the two molecules. 

However, IPES measurements for CoPc were not able to characterize the specific features of the 3d empty states, which, instead, are detected by linear-polarized Co L3 XAS. We aimed to shed light on these features of the central atom in the DOS measured by IPES, aiming at the definition of the empty orbitals’ sequence in CoPc overlayers. This question is not unambiguously addressed in the theoretical literature yet, as we will discuss. Moreover, we want to enlighten people about the electronic structure and empty state alignment of such anisotropic systems as CuPc and CoPc deposited on Au(110) 1 × 2.

The arrangement of almost unidirectional molecular architecture allows for different electronic dispersion along and orthogonal to the chains, which can have an impact on the charge injection and the anisotropic nature of transport properties in such nanopatterned systems. 

The high resolution of our IPES apparatus (the measured Fermi level from polycrystalline tantalum shows a resolution of 0.3 eV, see Figure S1) applied to CoPc and CuPc on Au(110) enables us to single out the 3d Co orbitals, for the first time, and to highlight the subtle details of the perturbation of MPc empty states (and their unoccupied frontier empty orbitals) involved in interaction with the Au substrate. We found that CoPc deposition induces a surface-driven molecular redistribution due to hybridization between the out-of-plane singly unoccupied CoPc orbital and the Au states, while the CuPc only slightly interacts with Au states. Moreover, we discuss a coherent description of the interface electronic structure of CoPc and CuPc, related to the distinct occupancy of the 3d of the metal ion in MPcs, complementary to previously published X-ray absorption research.

## 2. Materials and Methods

A detailed protocol for the MPc/Au(110) reproducible growth in the diverse apparatuses was defined at the Nanostructures at Surfaces laboratory at the Department of Physics of the Sapienza University in Rome [29]. The 1 × 2 Au(110) substrate was cleaned using standard procedures, using cycles of sputtering and annealing [4,12]. CoPc and CuPc powders were sublimated in ultra-high vacuum (UHV) from resistively heated quartz crucibles onto the clean substrate kept at 410 K, by using a rate of deposition of 0.5 Å/min. Reconstruction quality was revealed by LEED. One single layer (SL) corresponds to the nominal density of the MPc arranged in the (5 × 7) reconstruction for CoPc and (5 × 3) for the CuPc [5,6]. Coverages higher than the single layer are obtained by depositing MPc on clean gold surfaces at about 100 K.

Angular-resolved IPES measurements [30] were obtained in normal incidence (i.e., 90° between the electron impinging beam and samples’ surface, NI) by using a homemade Erdman–Zipf electron gun that can be rotated around the sample surface. Photons from the sample are revealed by a homemade Geiger–Mueller detector at 30° to the normal sample and built with a He-I_2_ gas mixture and a SrF_2_ entrance window filtering photons at hv = 9.5 eV. The data resolution results ≤ 300 meV, as measured by the Fermi level (E_F_) onset of a clean Ta foil (Figure S2 in SI). The spectra are normalized to the incident electron beam current. The electron beam divergence is better than 3°. The current density was below 2 × 10^−6^ A/cm^2^. To minimize the damage to the molecular layer during acquisition, the spectra were taken onto different freshly prepared layers and various areas of the samples, choosing a new area after about 10–15 min. The single scans were subsequently numerically summed. 

STM images were taken at the STM-UHV apparatus connected to the ID32 beamline of the European Synchrotron Radiation Facility (ESRF) in Grenoble, where also XAS measurements were carried out.

## 3. Results and Discussion

**Figure 1:**
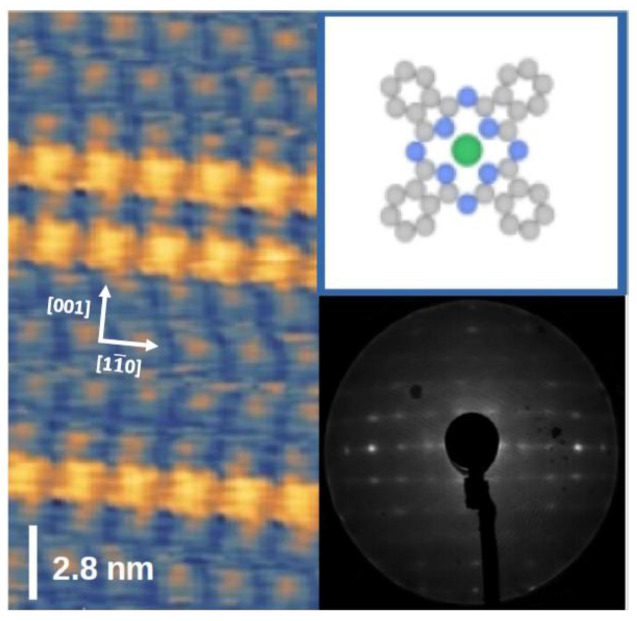
STM of the CoPc adsorbed on Au(110), nominal coverage 0.75 SL. The arrows indicate the gold direction of the surface missing row lattice cell. The image is taken at constant current (0.7 nA, −1 V bias) at room temperature (**left** panel): 5 × 5 self-assembled chains overall the topographic image, with a couple of overlying assembled chains, distant 2.82 nm, corresponding to a ×7 underlying gold lattice; LEED indicates the 5 × 5 reconstruction (**right-bottom** panel); sketch of the CoPc molecule, Co ion (green), N atoms (blue), C atoms (gray) (**right-top** panel).

The MPcs on the anisotropic 1 × 2 Au(110) surface are arranged in rows along the [1$\bar{1}$0] gold lines, forming ordered systems, as proven in the STM in Figure 1 (left panel). Those reconstructed channels include flat-lying parallel molecular chains. These are organized in ordered structures along the [001] direction for the specific coverage. The chains lead to a ×5 symmetry reconstruction in the sub-monolayer, as found in the LEED of Figure 1, right-bottom panel and STM (Figure 1, left panel). Further deposition of CoPc induces a ×7 periodicity of the chains of the single layer (SL), as discussed in detail in refs. [6,12], while GIXRD evaluation demonstrates that CuPc induces (5 × 5) and (5 × 3) reconstructions [5].

**Figure 2:**
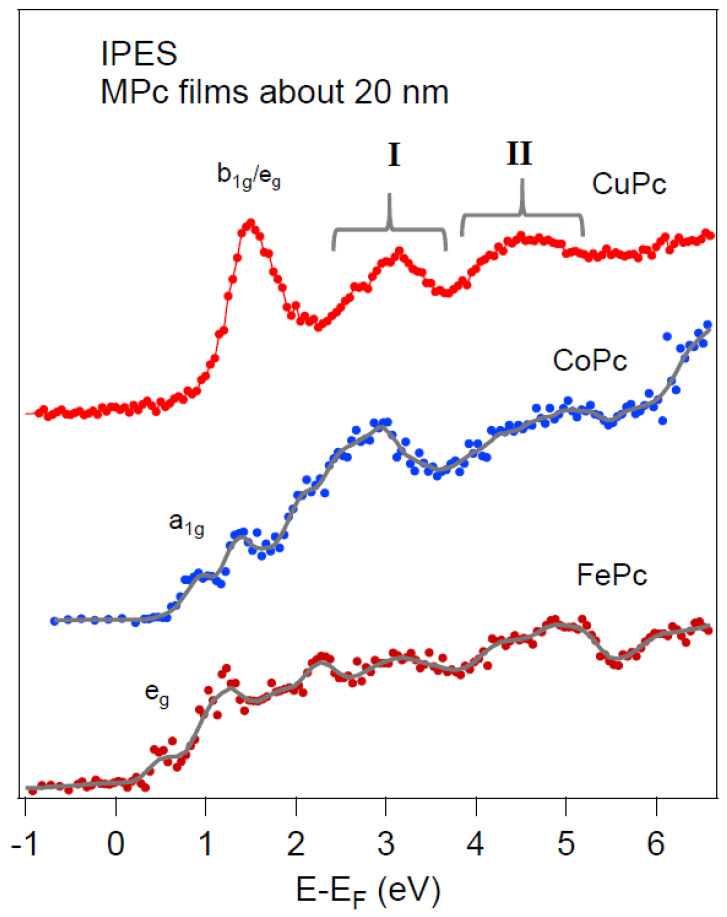
IPES spectra of α-structure MPc films deposited on the Au(111) surfaces. The first unoccupied state symmetry is labeled, regions labeled I and II are specific to the unoccupied orbitals of the MPc see the text for details.

We first discuss the empty spectral distribution obtained by IPES of MPc α-structured films as a reference (Figure 2) of CuPc (3d^9^), CoPc (3d^7^), and FePc (3d^6^) deposited on Au(111), highlighting the 3d orbitals’ contribution. MPc films present polymorphism [3], and the herringbone α-structure with the C2/c group can be grown onto Au(111) [31]. The distinct features of the IPES spectra labeled I and II above E_F_ are specific to the unoccupied orbitals of the MPc [16,21,24,27,32]. The spectra are formed through the superposition of empty orbitals positioned at the 3d metal atoms and of organic orbitals (i.e., with the contribution of C and N atoms, see Table S1 and Figure S1 in SI). IPES features below 3 eV are due to states originating from the mixing of 3d metal and ligand orbitals, while the dominant emission above 3 eV comes from pure organic empty orbitals.

The assignments and energies for the isolated MPc unoccupied states with 3d characteristics are still under debate in the theory literature, due to the impact of the density functional theory (DFT) used to model the exchange–correlation interaction on the theoretical density of states (see, for example, the discussion in references [33,34,35,36,37]). In any case, the 3d orbitals contribute to the frontier orbitals. Notably, in the case of solid-state aggregation of tetrapyrrole molecules (porphyrins and phthalocyanines), changes in the electronic properties can take place, as theoretically found in the β-structure of CoPc [38].

The IPES spectra in Figure 2 become more complex depending on the 3d electronic configuration (Table S1 in SI): the number of features increases with the decreasing central metal occupancy. The differences in the spectral features are ascribed to the different 3d metal configurations of the metal centers having the following: Cu^2+^ d^9^ and Co^2+^ d^7^. As mentioned before, both molecules have spin ½ and a single-occupied orbital. In the CuPc molecule, the SUMO is related to the b_1g_ state due to the partially occupied 3D_x2-y2_ state of Cu [5,39]. The CoPc has a more complex 3d electronic configuration, and the main accepted configuration assigns the single-occupied orbital to the d_z2_ out-of-plane state (a_1g_) of Co [3,6].

The FePc has an additional 3d hole and a spin = 3/2 (see Table S1 in SI).

The SUMO of CuPc b_1g_ is energetically close to the LUMO eg (with C and N contributions), mainly related to the pyrrole ring. This is confirmed by recent studies [16,20,24]. In the IPES pioneering reference [18], the LUMO presented a double feature that was assigned differently to e_g_ and b_1g_ molecular orbitals. Recently, this double feature has been proved to be induced by damage [27], with the prominent first feature being due to both b_1g_ and e_g_ states, in agreement with our present results.

IPES can be compared with XAS taken at different elemental edges, considering that XAS is due to the *local* joint density of electronic states through the transitions induced by radiation from the core level (1s in case of K edge and 2p in case of L_2,3_ edges) to the accessible empty states (according to the dipole selection rules) [16,40], and the final state of the system is influenced by core-hole effects. In any case, the highly structured L_3_ is related to the occupancy (see, for example, ref. [6]). In the figure, the first features above the EF in each MPc data are tentatively assigned to the frontier empty orbitals with 3D characteristics, following the sequence proposed in ref. [15] based on XAS L_3_ spectra. In the theoretical calculation of CoPc DOS (see previous discussion and refs. [33,34,35,36,37]), the sequence of the 3d empty states often found the eg closer to the E_F_, at variance of Co L3 XAS results that always found the a1g as the first empty state oriented orthogonal to the molecular plane, as discussed in the Introduction. This assignment is unambiguously due to the feature symmetry (p-polarization).

It is worth noting that with LUMO (Lowest Unoccupied Molecular Orbital), we mean the frontier *molecular* orbital, with contributions from C and/or N. Strictly speaking, this is not always the LUMO orbital in the MPcs series, because in some cases, part of the 3D orbitals of the central metal is unoccupied.

Concerning CoPc (3d^7^) thin-film IPES assignments, depending on the theoretical functional used, the frontier empty orbital is found either as the a_1g_ SUMO (purely Co 3d_z_^2^) or LUMO eg character. Recently combined PES-IPES measurements of CoPc/Au(111) thick films (25 nm) assigned the first empty state to the a1g unoccupied d_z2_ orbital [28]. The e_g_ symmetry state LUMOs are located at about 1.7 eV above E_F_.

Finally, FePc presents a spin = 3/2, and it shows a more complex distribution of features (Figure 2), with a peak at ~0.5 eV above E_F_, in agreement with recent IPES measurements [41].

**Figure 3:**
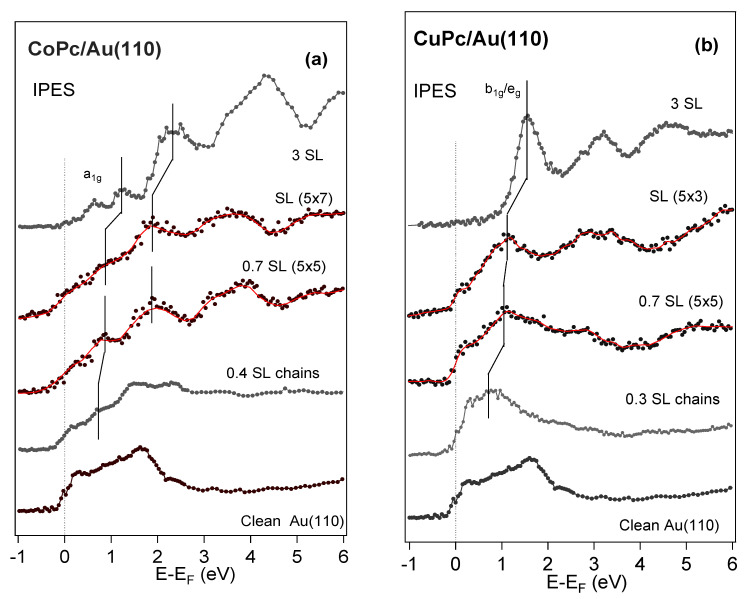
NI IPES spectra for different coverages of CoPc (**a**) and CuPc (**b**) on the Au(110) surface. Clean Au(110) and multilayer (3 SL) spectra are shown for comparison. Curves obtained by smoothing are shown by red lines. The first unoccupied state symmetry in both multilayer spectra is labeled.

We now focus on the MPc/Au(110) interfaces in the low-coverage regime. Figure 3 shows the IPES measurements as a function of CoPc (a) and CuPc (b) coverage; multilayer (3 single layers, 3SL) spectra are shown for comparison. The clean Au(110) NI spectrum is in agreement with references [42,43].

We observe remarkable differences between the two MPcs. We interpret this as due to the different orbital occupancy of the metal ions that lead to different interactions of the molecule with the underlying gold surface.

Upon increasing the MPc coverage, the empty molecular states emerge and are clearly detectable up to 3 ML, in agreement with the IPES spectral density measured for CuPc and CoPc films grown on Au(111) (Figure 2 and ref. [20]). It is worth mentioning that the energy positions to E_F_ of the pure organic empty orbitals are different for CoPc and CuPc, showing, again, the influence of the central metal on the energy alignment.

The IPES data of the CuPc/Au(110) system (Figure 3b) show that the emission close to EF is dominated by the metallic substrate, while the molecular state intensities become prominent at about 0.5–0.7 SL, corresponding to the (5 × 5)-symmetry reconstruction of the interface [12]. The emission at E_F_ of the underlying gold and the LUMO peak (labeled by the ticks in the figure) dominates the spectral emission. The energy shifts versus coverages of all the empty molecular states are influenced by the screening and the molecular orientation with the thickness of the adsorbed molecules [44]. 

Recently, STS results [45] indicated that the LUMO CuPc on Au(110), in a very low-coverage regime (≤0.3 SL), is always located above E_F_ for the Au surface, in agreement with our assignment at 0.3 SL and confirming the weak interaction, even for coverages below the molecular chain formation and a negligible substrate/molecule CT.

In the CoPc case, the IPES emission close to the E_F_ results considerably broadened after 0.4 SL of CoPc on Au(110), at the variance of the CuPc/Au(110) interface, as previously noted. The other empty orbitals present only minor changes (and a possible reduced energy separation) and can be related to the features present in the 3 SL spectra, though shifted closer to E_F_ [46]. The broadening of the E_F_ emission (from 0.3 eV to about 0.5 eV, as evaluated by a step function, not shown) for CoPc coverages up to the SL suggests a spread in the a_1g_ emission and a rehybridization with the underlying Au states.

At coverages above the SL, the state that we interpreted as a_1g_ emerges above the E_F_. This result is compatible with a Co^2+^ a_1g_ of those molecules that are not in contact with the Au substrate.

**Figure 4:**
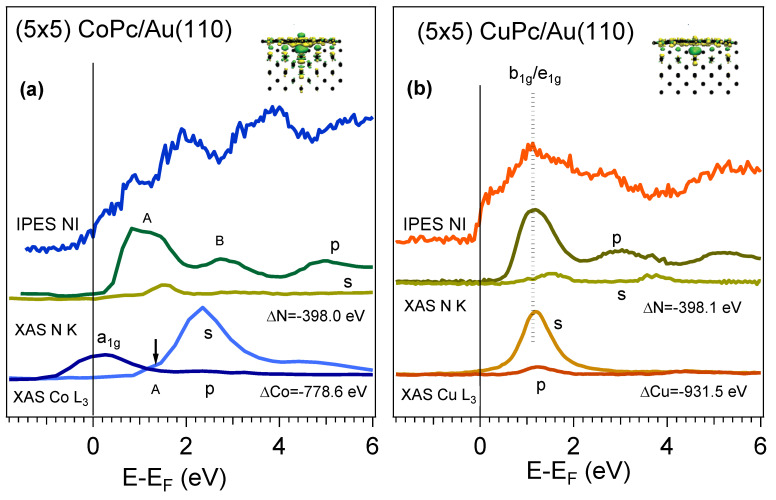
Linear polarized XAS measurements of N K and L_3_ metal edges [6] aligned to the NI IPES spectra features of (5 × 5) CoPc/Au(110) (**a**) and (5 × 5) CuPc/Au(110) (**b**). The shifted energies of the XAS edges, ΔN and ΔMetal, are indicated. The s and p labels indicate the polarization geometry. The double peak in the N K edge(labeled A) is due to the slightly different binding energies of the two no-equivalent N atoms group in the molecule. The contribution in IPES is a single peak, confirming the detection of the final state of the transition N1s → eg. The arrow in XAS Co L_3_ can be related to the Co-contribution of the eg orbital. In the inlets the interface positive (green) and negative (yellow) charge density transfer upon adsorption of the single molecule onto Au(110), in the two cases. See the text and ref. [6] for details.

Finally, to confirm our empty state assignments, we compare the NI IPES spectra of the (5 × 5) systems of MPc on Au(110), with the element-selective linear-polarized XAS results of the respective N and metal edges, Figure 4a,b. Curves labeled p (s) are taken in p-geometry (s geometry), i.e., with the impinging electric-field vector normal (parallel) to the plane of incidence. The insets report the charge densities upon the adsorption of single molecules on Au(110) in the two cases, as calculated by DFT (details in ref. [6]).

The linearly polarized radiation XAS data [6], taken in the two s- and p- acquisition geometries, show a strong dichroic effect due to the flat adsorption of the (5 × 5) (see Figure 1 and ref. [4]), with the p-geometry exciting the out-of-plane resonances (π*-related in the case of K edge) and the s-one exciting mainly the in-plane transitions (for example, the σ*-empty orbitals in K edge at higher photon energies, Figure S4 [15]). In this way, the a_1g_ state, which lies orthogonal to the molecular plane (Figure S1), is assigned.

For a neat assessment of the IPES features, the XAS data had been shifted, aligning the XAS with IPES peaks. The strength shift ΔE of XAS spectra was accomplished [24,47], neglecting the interplay associated with the hole induced by the photo-absorption, as discussed in refs. [16,40] (this factor is out of the scope of the present paper).

There is a straight one-to-one correspondence between the three main absorption π* resonances above the N K edge p-polarized XAS and the three higher-lying IPES structures above E_F_, thus confirming the prevalent organic nature of these IPES peaks, common to all MPcs. Peak A is related to the N → eg transition, commonly referred to as LUMO in the XAS literature; the broad B peak is called LUMO+1-LUMO+2 and includes the transitions associated with the b1g final orbital. It is worth noting that the double peak in the N K edges in both cases is due to the slightly different binding energies in the initial state of the transition (N1s → eg) due to the core level of the two no-equivalent N atom groups in the molecule. The contribution in IPES consists of a single peak, confirming the detection of the referred XAS final state of the transition.

We also notice that the s-polarized XAS shows a broad σ*-associated resonance at about 9 eV energy, also visible in the higher-lying IPES features (Figure S3).

At low energy, for the (5 × 5)-CoPc/Au(110) system, there is a clear p-polarized XAS feature at about 0.4 eV (in our alignment) associated with the a_1g_ state, with prevalent out-of-plane Co-d_z2_ contribution hybridized with the substrate metal state, as well as a peak at about 1.2 eV, associated with the e_g_ Co-ligand state (labeled by an arrow in Figure 4a). The charge density is high, corresponding to the out-of-plane direction. In the case of the (5 × 5)-CuPc/Au(110) system, there is the same clear correspondence of the N K absorption features with the main IPES structures, and the 1.2 eV peak presents a contribution of the b_1g_/e_g_ associated with the empty in-plane Cu-ligand states [46], the dichroism of the Cu L_3_ first transition is reversed in comparison to the Co L3 XAS spectra, and a lower out-of-plane charge density with respect to the CoPc adsorption, confirming a feeble CT between the Au substrate and CuPc [14].

Previous theoretical predictions suggest an intermixing between the a_1g_ state and gold, with a total quenching of the CoPc magnetic moment, as determined by XMCD investigation [6]. Thus, the IPES data confirm the hybridization of the a_1g_ empty molecular state, due to the charge density variation in the 3d Co^2+^ ion states interacting with the underlying metal, as shown in the theoretical evaluation in the inset of Figure 4a. It is worth noting that the reported calculations are performed for a single molecule on Au(110). Our results also confirm the intermixing of Co and Au for the unidirectional CoPc chains.

The observed dissimilarities in the interfacial interaction by IPES are also supported by photoemission spectroscopy [4,13]. In particular, a much higher chemical shift in the relevant core-level binding energies is detected for CoPc/Au(110) [13] than for CuPc/Au(110) [4] systems, indicating, again, that no significant CT takes place between CuPc and Au, while the CT is shown to be significant between CoPc and the Au substrate. The core-level binding energies of both the (5 × 5) MPc/Au(110) and thin-film systems are reported in Table S2. From these results, a clearly different behavior of the carbon and nitrogen energy levels is evident: in CoPc. They have opposite shifts (C up, N down) in comparison with the thin-film values, while in CuPc, they are both shifted at lower binding energies. Moreover, in CoPc, the Co 2p_3/2_ energy is highly changed. These results indicate a strong perturbation of the charge density redistribution in the CoPc chains, which is not simply due to screening, confirming the present work findings.

In summary, the CoPc adsorption on Au(110) induces a perturbation and a charge redistribution in the out-of-plane molecular orbital. The molecule–substrate interaction is controlled by mixing among the molecule and substrate electronic states, as stated and discussed in refs. [6,13]. The comparison of the molecular states intermixing using IPES and the element-selected empty DOS projection, as deduced by XAS, confirms our assignments. We stress here that this procedure is a powerful route to discovering and singling out the changes in the organic and metal centers in organic–inorganic interaction.

## 4. Conclusions

The ordered architectures of MPcs on the nano-template Au(110) surface are systems of interest in nano-devices, particularly due to the long-term stability of phthalocyanine molecular layers [13,48].

The electronic properties of organic overlayers strongly depend on interface morphology. The study of molecular-driven surface reconstruction is a key aspect to take into account for the transport engineering of novel devices.

In this work, IPES has been used to enlighten the molecule–substrate interaction for the self-assembled MPc adsorption on highly anisotropic gold, providing a coherent description of the interface electronic structure of CoPc and CuPc and the changes induced by the different occupancy of the 3d levels of the central metal ion, which is coherent with previously published results.

IPES data for the ordered CoPc and CuPc layers on the Au(110) surface, and element-selected XAS data taken at the same interfaces, allowed us to unravel the specific contributions to the empty states. In particular, orbital intermixing between Co-related states and Au has been revealed, while a weaker interaction with Au takes place for the Cu states. This high-energy-resolved IPES study underscores the subtle differences in the interaction at the interfaces of self-assembled MPcs chains on Au, depending on the central metal ion, and it can be a reference for further studies on the empty states in the investigation of organic molecular adlayers on surfaces.

## Data Availability

The data presented in this study are available in the article.

## Supplementary Materials

The SI can be downloaded at: https://www.mdpi.com/article/10.3390/nano14020158/s1, Table S1: Title The most probable electronic configuration of the MPc; Figure S1: Molecular orbitals in isolated MPc; Figure S2: Normal Incidence IPES spectrum of Ta polycrystalline film; Figure S3: Normal Incidence IPES spectrum of a CuPc film compared with the XAS data of N K and Cu L_3_ edges; Table S2: Photoemission core level binding energies. References [3,4,13,16,47] are cited in the supplementary materials.
