# Supplementary material for: Intermixing of Unoccupied States of Metal Phthalocyanine Chains Assembled on Au(110)
Source: Nanomaterials (Basel). 2024 Jan 11;14(2):158. doi: 10.3390/nano14020158 (PMC10819670; doi:10.3390/nano14020158)
Supplement: Supplementary file 1 [file nanomaterials-14-00158-s001.zip › nanomaterials-2746660-supplementary.pdf]

Supplementary data

Intermixing of Unoccupied States of Metal Phthalocyanine Chains Assembled on Au(110)

Abhishek Kumar [1,2,†], Maria Grazia Betti [3], Carlo Mariani [3], Manvendra Kumar [1,4], Pierluigi Gargiani [5], Cristian Soncini [1,2] and Maddalena Pedio [6,*]

[1] Istituto Officina dei Materiali-Consiglio Nazionale delle Ricerche, Basovizza SS-14, Km 163.5, 34012 Trieste, Italy; abhishek.e9363@cumail.in (A.K.); kmanav@gmail.com (M.K.); cristian.soncini@ifn.cnr.it (C.S.)
[2] Department of Physics, University of Trieste, 34127 Trieste, Italy
[3] Dipartimento di Fisica, Università di Roma "La Sapienza", Piazzale Aldo Moro 5, 00185 Roma, Italy; maria.grazia.betti@roma1.infn.it (M.G.B.); carlo.mariani@uniroma1.it (C.M.)
[4] Department of Physics, Institute of Science, Shri Vaishnav Vidyapeeth Vishwavidyalaya, Ujjain Road, Indore 453111, India
[5] ALBA Synchrotron Light Source, Carrer de la Llum 2-26, 08290 Barcelona, Spain; pgargiani@cells.es
[6] Istituto Officina dei Materiali-Consiglio Nazionale delle Ricerch, V. A. Pascoli s.n.c., 06123 Perugia, Italy
* Correspondence: pedio@iom.cnr.it
† Current address: Department of Physics, University Institute of Sciences, Chandigarh University, Mohali 140413, India.

1-3d occupancy in isolated MPc

The phthalocyanine is a square planar molecule belonging to the D_{4h} space group and coordinating the transition metals (M). Its complexated isolated form has an extended aromatic system, and a set of localized metallic states, 3d orbitals which split into three singlets (d_{z2}; d_{xy}; d_{x2xy2} with symmetry a_{1g}; b_{2g}; b_{1g} respectively) and one doublet (d_{xz}; d_{yz} with symmetry eg). By mixing with ligand orbitals of analogous symmetry, they form a set of molecular orbitals with metallic character (S2, a), while the ligand orbitals with incompatible symmetry remain localized on the organic portion of the molecule (S2, b).

Table S1 The most probable electronic configuration of the MPc.

Molecule	Occupancy	Electronic conguration
FePc	3d6	$b^2_{2g}\ e^3_g\ a^1_{1g}$
CoPc	3d7	$b^2_{2g}\ e^4_g\ a^1_{1g}$
CuPc	3d9	$b^2_{2g}\ e^4_g\ a^2_{1g}\ b^1_{1g}$

The Metal 3d orbitals energies depend on the occupation of the central metal ion. For discussion see Ref. [1].

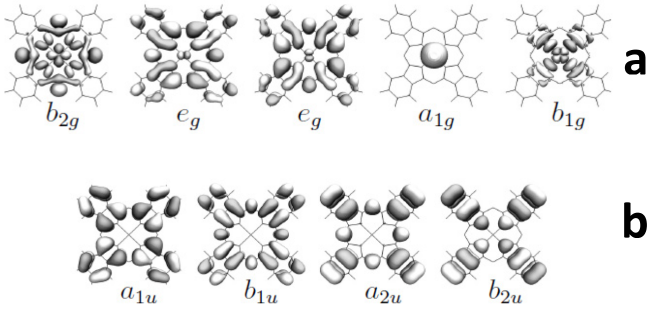

Figure S1 Molecular orbitals of isolated MPc and their symmetries in the D_{4h} group with metallic (a) and pure organic (b) character.

2 Determination of the IPES energy resolution

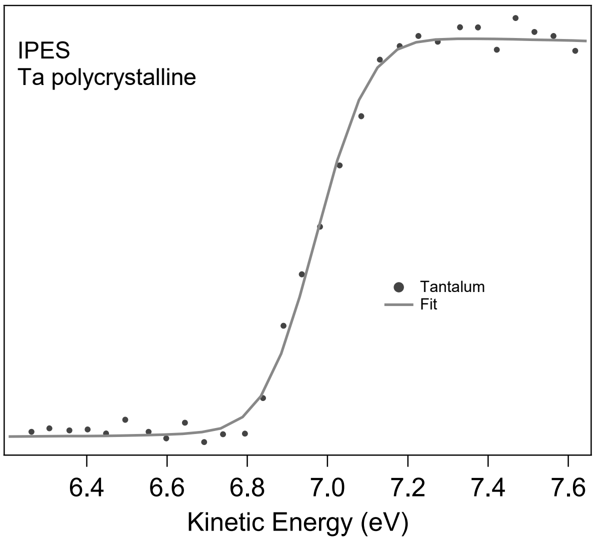

Figure S2 Normal Incidence IPES spectrum of Ta polycrystalline film. The fit is obtained by using a step function. The overall energy resolution results < 0.30 eV. All the IPES measurements in the text are referred as the Fermi level (E_F).

3 Example of alignment XAS-IPES

The features in IPES can be compared with XAS taken at the different elemental edges, taking in mind that XAS reflects the local density of the transition induced by radiation from the core level (1s in case of K edge and 2p in case of $L_{2,3}$ edges) to the empty states [2]. The final state of the system presents a hole in the core state.

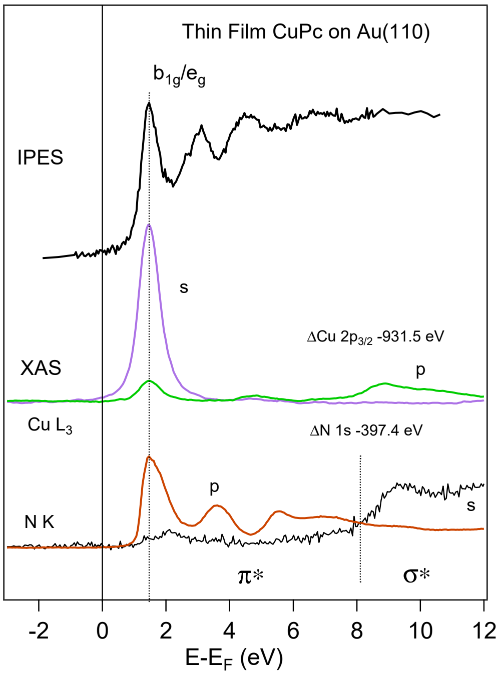

Figure S3 Normal Incidence IPES spectrum of a CuPc film compared with the XAS data of N K and Cu L_3 edges, shifted following the procedure of Ref. [2].

Figure S3 shows the linear polarized XAS-IPES comparison example in case of CuPc thin film. The p and s geometry spectra are labelled p and s, respectively. The XAS spectra have been aligned following Ref. [2]. The CuPc molecule presents a single 3d hole in the b_{1g} orbital associated with a Cu-d_{x2-y2} state extending in the plane of the molecule. The L_3 Cu edge presents a peak at 932.5 eV with a definite dichroic effect in p and s acquisition geometries associated with a transition to the b_{1g} and the e_g hybridized with the N-$p_{x,y}$ orbitals. The N K edge spectra show a similar dichroism. The regions of transition involving the and orbitals are delimited by a line at about 8 eV in the figure. The double peak in N K edge is due to the slightly different binding energies of the two no-equivalent N atoms group in the molecule.

4 Photoemission core level binding energies.

	**CoPc Thin films** EB (eV)	**5x5 CoPc/Au(110)** EB (eV)	**CuPc Thin films** EB (eV)	**5x5 CuPc/Au(110)** EB (eV)
Metal 2p $_{3/2}$	-780.2	-778.6	-935	-935
N1	-398.6	-399.1	-399.2	-398.7
N2	-399.0	-398.7	-398.9	-398.4
CB	-284.3	-284.1	-285.3	-285.8
Cp	-285.8	-285.3	-284.9	-284.4
Peak of the Empty Frontier Orbital	0.7	Spread between 0-0.5	1.5	1.1

Table S2 Comparison of the core levels binding energies of the two (5x5) MPc/Au(110) systems and the respective multilayers, from Refs. [4] and [5]. The peak of the first empty frontier orbitals are taken from Normal Incidence IPES spectra (this work).

References

1 Bartolomé, J.; Monton, C.;· Schuller, I.K. Chapter 9 Magnetism of Metal Phthalocyanines. In J. Bartolomé et al. (eds.), Molecular Magnets, NanoScience and Technology, Springer-Verlag Berlin Heidelberg, 2014, pp. 221-245. DOI 10.1007/978-3-642-40609-6_9.

2 Cook, P. L.; Yang, W.; Liu, X.; García-Lastra, J.M.; Rubio, A.; Himpsel, F. J. Unoccupied states in Cu and Zn octaethyl-porphyrin and phthalocyanine. J. Chem. Phys., 2011, 134, 204707, pages 1-7. DOI: 10.1063/1.3592937.

3 Gargiani, P.; Angelucci, M.; Mariani, C.; Betti, M. G. Metal-phthalocyanine chains on the Au(110) surface: Interaction states versus d-metal states occupancy. Physical Review B 2010, 81, 085412. Pages 1-7. DOI: 10.1103/PhysRevB.81.085412.

4 F. Evangelista, A. Ruocco, R. Gotter, A. Cossaro, L. Floreano, A. Morgante, F. Crispoldi, M. Betti, and C. Mariani, Electronic states of CuPc chains on the Au(110) surface. J. Chem. Phys. 2009, 131, 174710. DOI: 10.1063/1.3257606.

5 Massimi L, Angelucci M, Gargiani P, Betti MG, Montoro S, Mariani C. Metal-phthalocyanine ordered layers on Au(110): Metal-dependent adsorption energy. J Chem Phys. 2014, 140, 244704, 8 pages. DOI: 10.1063/1.4883735.
